# Table of Contents and Acknowledgements

**DOI:** 10.1080/26410397.2025.2595813

**Published:** 2026-01-23


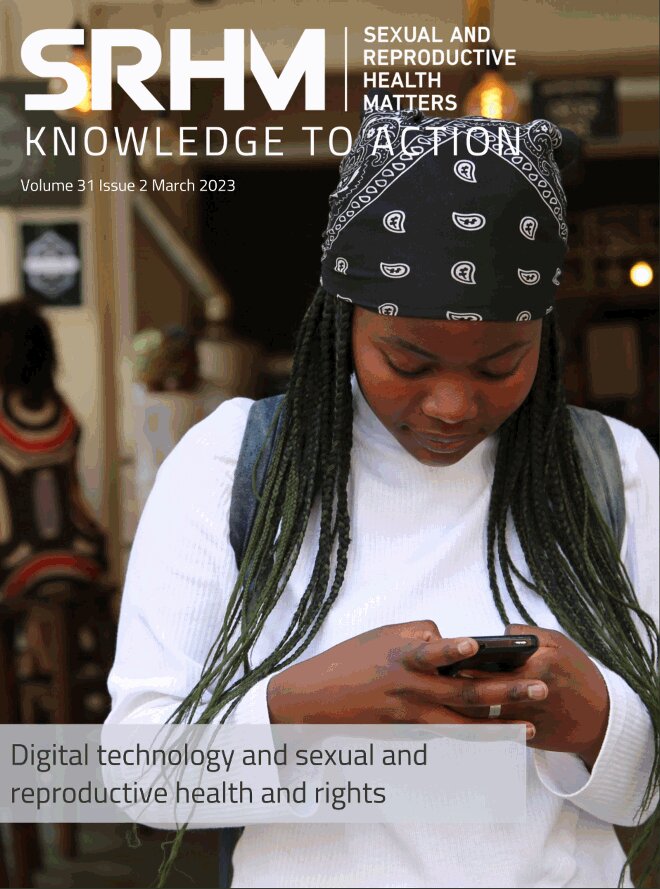


**Table:** 

***EDITORIAL***	
*Priya Nanda, Nina Sun, Emma Pitchforth, Eszter Kismödi*	Digital justice and rights: bridging the fault lines in digital technology and SRHR
***PERSPECTIVE***	
*Emily McMahon, Tamara Fetters, Nadia Lobo Jive, Mike Mpoyi*	Perils and promise providing information on sexual and reproductive health via the Nurse Nisa WhatsApp chatbot in the Democratic Republic of the Congo
***COMMENTARIES***	
*Kath Albury, Zahra Stardust, Jenny Sundén*	Queer and feminist reflections on sextech
*Bridget B Kelly, Maniza Habib*	Missed period? The significance of period-tracking applications in a post-*Roe* America
*Rajat Khosla, Vidisha Mishra, Sagri Singh*	Sexual and reproductive health and rights and bodily autonomy in a digital world
*Claudia A Lopes, Arthur Saitabau, Niharika Rustagi, Rajat Khosla*	A digital health governance agenda for sexual and reproductive health and rights
*Génesis Luigi-Bravo, Ana Ramirez, Caitlin Gerdts, Roopan Gill*	Lessons learned from developing and implementing digital health tools for self-managed abortion and sexual and reproductive healthcare in Canada, the United States, and Venezuela
***RESEARCH ARTICLES***	
*Joseph Adu, Matthea Roemer, Georgina Page, Elymas Dekonor, George Akanlu, Chris Fofie, Mary Teye, Patricia Opoku Afriyie, Adjeiwa Akosua Affram, Fidelia Ohemeng, Philip Teg-Nefaah Tabong, Duah Dwomoh*	Expanding access to early medical abortion services in Ghana with telemedicine: findings from a pilot evaluation
*Boladé Hamed Banougnin, Elona Toska, Brendan Maughan-Brown, William Rudgard, Lucas Hertzog, Janina Jochim, Alice Armstrong, Lucie Cluver*	Associations of social media and health content use with sexual risk behaviours among adolescents in South Africa
*Juliana Friend*	Digital privacy is a sexual health necessity: a community-engaged qualitative study of virtual sex work and digital autonomy in Senegal
*Amnesty LeFevre, Olivia Welte, Kearabetswe Moopelo, Nicki Tiffin, Gaolatlhe Mothoagae, Nobukhosi Ncube, Nasiphi Gwiji, Manape Shogole, Amy L Slogrove, Noma Moshani, Andrew Boulle, Jane Goudge, Frances Griffiths, Lee Fairlie, Ushma Mehta, Kerry Scott, Nirvana Pillay*	Preferences for onward health data use in the electronic age among maternity patients and providers in South Africa: a qualitative study
*Joan Njagi*	A qualitative approach to interrogating the age and gender divide in digital SRHR platforms in Kenya
*Julius Njogu, Grace Jaworski, Christine Oduor, Aarons Chea, Alison Malmqvist, Claire W. Rothschild*	Assessing acceptability and effectiveness of a pleasure-oriented sexual and reproductive health chatbot in Kenya: an exploratory mixed-methods study
*David Aduragbemi Okunlola, Oluwatobi Abel Alawode, Abayomi Folorunso Awoleye, Benjamin Bukky Ilesanmi*	Internet use, exposure to digital family planning messages and sexual agency among partnered women in Northern Nigeria: a cross-sectional analysis of the 2018 Nigeria Demographic and Health Survey
*Nora Piay-Fernández, Emma Stenbacka, Mariana Calderón Jaramillo, Giovanni Guerrero, Alejandro Antonio Solano Rodríguez, Paola Montenegro, Diana Carolina Moreno, Amanda Cleeve*	Implementing medical abortion through telemedicine in Colombia: a qualitative study
*Nirvana Pillay, Nobukhosi Ncube, Kearabetswe Moopelo, Gaolatlhe Mothoagae, Olivia Welte, Manape Shogole, Nasiphi Gwiji, Lesley Scott, Noma Moshani, Nicki Tiffin, Andrew Boulle, Frances Griffiths, Lee Fairlie, Ushma Mehta, Amnesty LeFevre, Kerry Scott*	Translating the consent form is the tip of the iceberg: using cognitive interviews to assess the barriers to informed consent in South African health facilities

**Table:** 

**Executive Editor:** Emma Pitchforth **Chief Executive**: Eszter Kismödi **Senior Editors:** Sarah Keogh, TK Sundari Ravindran **Managing Editor:** Pete Chapman **Monitoring Editor**: Pathika Martin **South Asia Hub Manager**: Sanjeeta Gawri **Communications Manager**: Alexane Bremshey/Jessica MacKinnon **Operations Manager**: Amy Guthrie **Associate Editors:** Laura Ferguson, Atsumi Hirose, Nambusi Kyegombe, Helen Potts, Mindy Jane Roseman, Nina Sun, Joyce Wamoyi **Peer reviewers:** Taofeekat Oluwatosin Adigun, Thierry Almont, Hazal Atay, Catherine Atkin, Abul Hasan BakiBillah, Emily R. Boniface, Eoghan Brady, Leona Cilar Budler, Fiona de Londras, Dulce Ferraz, Edson Santos Ferreira-Filho, Anna Fiastro, Emmalee Ford, Veronica L. Gregorio, Alexander Suuk Laar, Megan S C Lim, Farrah Madanay, Francis Masiye, Emily McMahon, Ruth Bonareri Momanyi, Jessica E. Moulton, Jennie Pearson, Adrienne Pichon, Monika Pobiruchin, Regina M Renner, Maria Santaguida, Nicola Sheeran, Maia Sieverding, Sarita Sonalkar, Christina Sun, Signe Svallfors, Tigest Tamrat, Robert Terry, Hua Wang, Alice Welbourn, Teng Zhao **Funding acknowledgement:** We are grateful to the following for supporting publication of this issue: Bill & Melinda Gates Foundation DKT International United Nations University	**Cover image:** Angelo Moleele. Woman standing while holding black phone. Published on July 4, 2018. Johannesburg. Free to use under the Unsplash License **Translation:** Françoise de Luca-Lacoste translated abstracts from English to French and Lisette Silva translated abstracts from English to Spanish. **Copyright © 2026 Sexual and Reproductive Health Matters**. This is an Open Access journal distributed under the terms of the Creative Commons Attribution License (http://creativecommons.org/licenses/by/4.0/), which allows for sharing and adapting the work for any purpose, even commercially, provided appropriate credit is given with a link to the originally published item, a reference to the author(s) and links to their homepages, reference to the license under which the article is published and a link to this, as well as an indication of any changes that have been made to the original. ISSN (Online) 2641-0397.

www.srhm.org / www.srhmjournal.org

@SRHMJournal

